# Workaholism and work-family conflict among critical care nurses: a cross-sectional study

**DOI:** 10.1186/s12912-025-03465-3

**Published:** 2025-07-03

**Authors:** Azza Hassan Mohamed Hussein, Lamiaa Zakria Taha Ramadan, Amal Diab Ghanem Atalla

**Affiliations:** 1 Department of Nursing Administration, Faculty of Nursing, Alexandria University, Alexandria, Egypt; 2 Head Nurse at Borg El Arab Hospital, Alexandria, Egypt

**Keywords:** Nurses, Workaholism, Work-family conflict, Critical care

## Abstract

**Background:**

Critical care nurses’ boundaries between personal and professional life are sometimes blurred by the high demands placed on nurses. The rise in workaholism in this high-stress setting puts nurses’ health at serious risk and can intensify work-family conflict, endangering both personal health and well-being and professional output.

**Aim:**

This study examined the relationship between workaholism and work-family conflict among critical care nurses.

**Design:**

A descriptive cross-sectional design that adheres to STROBE criteria.

**Methods and tools:**

The study participants consisted of 360 nurses from the critical care units at Alexandria University Hospital. This hospital is the highest-capacitated hospital in Alexandria governorate in terms of bed capacity (6760), number of nurses, and the diversity of services rendered in different qualifications. It provides therapeutic and educational services. Nurses completed two tools, the Dutch Work Addiction Scale (DUWAS) and the Work-Family Conflict Multidimensional Scale (WFC). Statistical tests comprised the Pearson coefficient, the Student t-test, and a one-way ANOVA. The 5% level was used to assess the results’ significance.

**Results:**

Overall workaholism (DUWAS) is positively and significantly correlated with WFC (*r* = 0.415, p = < 0.001).

**Conclusion:**

The results of this cross-sectional study show that among critical care nurses, workaholism is a major factor in work-family conflict. The necessity of focused organizational initiatives in healthcare settings is highlighted by these findings. To lessen excessive job participation, hospital administrators should specifically develop structured work-life balance initiatives, such as flexible scheduling, workload management guidelines, and access to mental health services. Recognizing workaholism symptoms and promoting helpful supervisory techniques should also be emphasized in leadership training.

**Clinical trial number:**

Not applicable.

## Introduction

### Critical care nurses must contend with escalating

 and intricate pressures stemming from workplace challenges while adapting to swift changes. The substantial workload necessitates extended hours and increased responsibilities, perpetually leading to the emergence of potential workaholic individuals [1, 2]. Professional life provides individuals with various experiences, including problem-solving, collaboration, recognition, and career advancement. In the current work environment, dedication to the organization and job, along with a strong work ethic, are increasingly viewed as desirable traits by employers. Workaholism can lead to numerous cognitive and behavioral effects that impact both an individual’s professional and personal life [3]. Workaholics often struggle to fulfill family expectations due to their inclination to prioritize work, leading to a correlation between workaholism and work-family conflict (WFC), which can create adverse circumstances. Family and job are important facets of a person’s life, and achieving a balance between the two is becoming progressively challenging.

## Literature review

### The concept of workaholism

Workaholism has been explored in various dimensions within the literature. Oates originally used the word in 1971, and characterized it as an ‘addiction to work,’ describing a compulsive and irresistible desire to participate in work continuously [4]. A maladaptive pattern of excessive job participation, workaholism, is defined in this study as an uncontrollable inner need to work, frequently at the detriment of personal and family life. Regardless of the need or benefits from outside sources, it indicates both excessive and obsessive working [4].

The compulsive characteristics of workaholism, along with the resulting lifestyle imbalance, rather than the significant time investment required, may represent the primary maladaptive aspect. Those who identify as “workaholics” or who others perceive as such are frequently compulsive workers who do not derive pleasure from their work, which results in a greater imbalance between work and family and lower life satisfaction than “positively engaged workers” or “unengaged workers.” [5].

### Work-family conflict

A form of inter-role conflict that results from the incompatibility of demands from both the work and family domains is known as work-family conflict (WFC). When work commitments make it more difficult to satisfy family obligations, or vice versa, this conflict arises. A type of inter-role conflict known as work-family conflict (WFC) occurs when the expectations of the family and job are incompatible, making it harder to fulfill one because of the obligations of the other. WFC may show up as behavior-, strain-, or time-based conflicts [6, 7].

The term “Work Interference with Family” (WIF) describes how work-related obligations negatively affect a person’s capacity to fulfill obligations to their family. This feature draws attention to circumstances in which work obligations, time restraints, or stress have a detrimental impact on family life and reduce the capacity to participate in family activities. On the other hand, when family commitments interfere with job requirements, this is known as Family Interference with job (FIW). This covers situations in which providing care, raising children, or performing other family responsibilities impairs commitment, attendance, or job performance. When FIW occurs, it may lead to increased absenteeism, worse job performance, or greater stress at work [6].

The relationship between family and work is not one-sided. Because work can interfere with family life and vice versa, it is said to be bidirectional. One effect of family workaholism is work-family conflict [7].

### Theoretical background

Workaholism is a multifaceted issue that may be linked to fundamental psychological needs for autonomy, competence, and relatedness, which are considered the driving forces behind human behavior. This condition can be associated with these needs; for instance, an individual who feels inadequate may exert excessive effort to achieve a sense of competence, particularly if this drive is of high importance to them. Consequently, the internal compulsion or fixation on work can be tied to unmet needs that individuals attempt to satisfy through their professional endeavors. In this context, it has been observed that a compulsive work ethic correlates with unfulfilled psychological needs [8]. The idea of workaholism seems to have significant theoretical similarities with related concepts like obsessive love for work and work over-involvement [9].

The Job Demands-Resources (JD-R) model, which offers a thorough framework for comprehending how job features lead to stress and conflict, serves as the foundation for this investigation. Workplace factors are divided into job demands (like workload and emotional strain) and job resources (like autonomy and support) by the JD-R model. These elements interact to affect employee well-being and outcomes like work-family conflict (WFC). Excessive job expectations in the high-stress field of critical care nursing can lead to maladaptive coping strategies, including workaholism, which is defined as an insatiable desire to work excessively. Even though workaholism could seem like a high level of participation at first, it frequently leads to psychological stress and depleted resources, which can result in WFC [10].

Furthermore, the Conservation of Resources (COR) hypothesis lends credence to this perspective by arguing that stress is mostly caused by the loss of valued resources, such as time, energy, and emotional capacity, which people try to preserve and safeguard. The likelihood of conflict between work and family duties increases when nurses overextend themselves owing to workaholic tendencies, depleting their personal and familial resources. The JD-R model provides a clear lens through which to view occupational characteristics that influence nurses’ work-life balance by framing workaholism as a maladaptive reaction to job expectations and interpreting WFC as a strain outcome from resource depletion. The results of the study can be interpreted and focused treatments can be developed with the help of this theoretical integration [11].

### Significance of the study

The current body of research on workaholism in healthcare professionals has mainly concentrated on physicians, managers, and general nursing staff, while critical care nurses have received insufficient attention. These nurses are essential to the healthcare system, tasked with delivering intricate, high-acuity care to critically ill patients. Nevertheless, the extent of workaholism within this specialized group remains largely unexamined. This study seeks to fill this important research void by evaluating the prevalence of workaholism among critical care nurses.

This research is important because it clarifies the intricate connection between workaholism and work-family conflict among critical care nurses, a population that is especially susceptible to stress and burnout because of the demanding nature of their jobs. This study offers important insights on how excessive work involvement affects their personal and family lives, which can help nursing leaders, hospital administrators, and legislators create focused measures to support a better work-life balance [4]. In addition to improving nurses’ well-being and job satisfaction, tackling these issues is crucial for improving organizational sustainability and the quality of patient treatment in critical care settings [4–6].

#### Aim of the study

The purpose of this study is to investigate the connection between critical care nurses’ work-family conflict and workaholism.

#### The research question is

What is the relationship between workaholism and work-family conflict among critical care nurses?

## Methods

### Study design

Following STROBE principles, a cross-sectional descriptive design was used.

### Setting

All critical care units at Alexandria Main University Hospital participated in the study (*N* = 23). This hospital is the highest-capacitated hospital in Alexandria governorate in terms of bed capacity (6760), number of nurses, and the diversity of services rendered in different qualifications. It provides therapeutic and educational services, in addition to its role in scientific research, and provides public non-paid health services and patient care to different specialists. It is the first university hospital to take significant action to meet the General Authority for Health Accreditation and Regulation’s (GAHAR) criteria.

Critical care units were specifically selected as the study setting because of the high patient acuity, quick decision-making, and ongoing exposure to life-or-death circumstances that these settings present, all of which put an exceptional amount of strain on nurses. Compared to nurses in other disciplines, critical care nurses are more vulnerable to workaholism and its related effects, including work-family conflict. The study intends to provide more accurate and significant insights that can direct treatments catered to the requirements of critical care personnel by concentrating on this environment to capture the distinct pressures and dynamics that lead to unbalanced work patterns.

### Sampling

Using G*Power (version 3.1.9.7) for statistical power analysis, a sample size of 344 participants was deemed sufficient out of a total population of 415 nurses employed in critical care units. A medium effect size (f² = 0.15), a significance level (α) of 0.05, a statistical power (1–β) of 0.95, and the inclusion of up to five predictors (such as workaholism dimensions and demographic variables) in the main analysis were the criteria used to calculate power. This sample size was thought to be adequate for highly confidently identifying significant associations in correlational or multiple regression studies. The sample size was slightly expanded to 360 nurses to account for potential problems such as incomplete questionnaires, inconsistent or repeated responses, or unanticipated participant withdrawal.

To guarantee representation across the various critical care units (such as the intensive care unit, critical care unit, emergency room, and recovery unit) within the participating institutions, a proportional stratified random sample technique was employed. Computer-generated random numbers were used to pick eligible participants at random from staff rosters within each stratum. Criteria for Inclusion: Full-time critical care nurses who have worked on their present unit for at least six months.

After being briefed on the study’s objectives and confidentiality protocols, nurses willingly consented to take part. Nurses who have worked in critical care units for fewer than six months are excluded. nurses who, at the time of data collection, were on administrative duty, sick leave, or extended leave. responses that are invalid or incomplete (such as several answers to single-response questions or significant missing data).

### Ethical considerations

Every technique used in this research followed the appropriate guidelines offered in the Declaration of Helsinki (DoH-Oct2008). The Alexandria University Research Ethics Committee’s nursing faculty approved the study protocol (IRB00013620 (9/19/2025). Before consent, nurses were kept aware of the study’s purpose. To maintain confidentiality and anonymity, each questionnaire was given a code number. As promised to nurses, data use was limited to research. The ability to withdraw from the study has been verified.

### Tools

#### Tool I. The Dutch work addiction scale (DUWAS)

Using ten elements, it was created by Schaufeli et al. (2009) and used by the present study to gauge nurses’ workaholism [12]. Working excessively (WE) and working compulsively (WC) are two 5-item scales that are evaluated by these 10 items. A 4-point Likert scale, with 1 denoting “rarely” and 4 denoting “almost always,” was used to rate each item. These items are separated into two scales, Working Compulsively (WC) and Working Excessively (WE), each with five items. ‘Rarely’ (1) to ‘nearly usually’ (4) were the extremes of the 4-point Likert scale used to quantify responses. For both scales, a cutoff score of 2.5 was set. Workaholism was indicated by scores above 2.5 on both WE and WC, whereas hard work was indicated by scores above 2.5 on WE and below 2.5 on WC. Conversely, scores below 2.5 on WE and above 2.5 on WC identified a compulsive worker, and scores below 2.5 on both scales classified individuals as relaxed workers. The reliability of the internal consistency of the workaholism construct was confirmed with a Cronbach’s alpha coefficient of α =.78 and ω =.78, demonstrating acceptable internal consistency [13].

#### Tool II. The Work-Family conflict multidimensional scale (WFC)

It is a standardized scale created by Carlson et al. (2000), and the present researcher will use it to evaluate nurses’ conflicts with work and home pressures [14]. 18 items make up the scale, which evaluates six aspects of work-family conflict (WFC). Together, the three forms of WFC time-, strain-, and behavior-based, and their two directions, Work Interference with Family (WIF) and Family Interference with Work (FIW), give rise to these dimensions. Time-based WIF, time-based FIW, strain-based WIF, strain-based FIW, performance-based WIF, and performance-based FIW are among the specific dimensions. For measuring, a five-point Likert scale was used, with 1 denoting strongly disagree and 5 denoting strongly agree. Coefficient alpha was used to assess internal consistency for each of the six dimensions, and reliability scores for time-based WIF (5.87), time-based FIW (5.79), strain-based WIF (5.85), strain-based FIW (5.87), behavior-based WIF (5.78), and behavior-based FIW (5.85) exceeded the generally recognized cutoff of.70 (Nunnally, 1978) [14].

The researcher also created a professional and sociodemographic data sheet that included the respondents’ age, gender, marital status, current unit, years of nursing, unit experience, educational level, and shift work.

### Pilot study and reliability

Instead of the participants chosen for the primary study, a pilot study was carried out with 10% of the nursing staff (*n* = 36). This was carried out to predict the amount of time needed to finish the questionnaires, identify potential difficulties during data collection, and evaluate the instruments’ clarity, application, and viability. The instruments were finalized in their original forms with no changes based on the pilot study’s findings.

### Tools validity and reliability

To conform to Egyptian cultural norms, the instruments were translated from English to Arabic and then back-translated. Two certified translators with degrees in translation and previous nursing and healthcare expertise were selected to translate the tool from English to Arabic on their own. A single Arabic version of the surveys was produced by combining the completed translations of each translator, who worked individually. Two additional bilingual translators with experience in healthcare and nursing translation were then given the combined Arabic version, and they translated it back into English, creating a single back translation. Every item was thoroughly checked for inconsistencies once the two language versions were prepared, and those that were discovered were modified to remain true to the original English version.

A variety of techniques, such as Cronbach’s alpha, corrected item-total correlations, exploratory factor analysis (EFA), confirmatory factor analysis (CFA), content validity, and inter-dimensional correlations, were used to assess their validity and reliability. The instruments’ content validity was evaluated by a panel of seven subject-matter experts, comprising four lecturers and three professors from Alexandria University’s Faculty of Nursing. WFC’s content validity index was 0.972, whereas DUWAS’s content validity value was 0.940. The reliability and internal consistency of the instruments were assessed using the Cronbach’s alpha coefficient. According to the results, the instruments were highly reliable; DUWAS and WFC both recorded Cronbach’s alphas of 0.833 and 0.826, respectively.

### Overcame the problem of common method biases

The researchers implemented a combination of statistical controls and procedural techniques to tackle the problem of common method bias (CMB). To reduce social desirability bias and promote truthful answers, they ensured the anonymity and confidentiality of participants. Furthermore, to diminish the likelihood of respondents providing identical answers across multiple items, they provided clear instructions and a variety of response formats while segmenting the questionnaire’s key metrics. The researchers also carried out a preliminary study with 10% of the population to improve the questionnaires, guarantee clarity, and reduce ambiguity that might lead to CMB. The authors successfully reduced the possible influence of common technique bias on the findings of their investigation by incorporating these tactics.

### Data collection

Questionnaires were hand-delivered to each participant in their separate work environments throughout morning, evening, and night shifts to collect data from the nursing staff. The goal of the study was explained to each nurse for around 10 min, after which the required instructions were given. Both general directions on how to fill out the questionnaires and detailed instructions for each item were given. After carefully reading each statement, nurses were asked to select the response that best reflected their opinions. Each nurse was told to return the three completed questionnaires to the researcher. Early April 2024 marked the start of the three-month data collection phase, which ended at the end of July 2024.

### Statistical analysis

Using IBM’s Statistical Package for Social Science (SPSS) software version 23.0, the gathered data was statistically analyzed and presented in tables, graphs, and charts. When comparing more than two categories, a one-way ANOVA test was used. The student t-test was used to compare two groups with quantitative data that was regularly distributed. The Pearson correlation coefficient was used for the analysis of quantitative data that did not fit a normal distribution. Means, standard deviations, medians, ranges, and percentages were among the descriptive statistics that were calculated. The means of regularly distributed data were compared using independent t-tests for continuous variables, the median differences of non-normally distributed data were evaluated using Mann-Whitney U tests, and categorical data were compared using a chi-square test.

A p-value of less than 0.05 was deemed statistically significant. The internal consistency of the scales was assessed using Cronbach’s α coefficient.

## Results

### Sociodemographic and professional characteristics of the nurses studied

Table 1 showed that 40% of the studied nurses were in the age group from 20 to less than 30 years, and 10.8% of the studied nurses were more than 50 years, with a mean age of 34.4 ± 9.6. Most of them (82.5%) were female, and more than half were married (57.8%), whereas about one third (33.8%) were single. According to the level of education, 38.6% of the nurses studied had a Bachelor of Nursing degree, while more than one-fourth (26.7%) had a health-associated degree. About one-fifth of the nurses studied (20.8%) worked in ICUs, and 8.9% and 8.6% worked in the Anesthesia and toxicology departments, respectively, whereas only 0.8% worked in the pediatric department.

**Table 1:** Sociodemographic and professional characteristics of the studied nurses (*N* = 360)

Demographic characteristics	No.	%
**Age (years)**		
20-	144	40.0
30-	114	31.7
40-	63	17.5
≥ 50	39	10.8
Mean ± SD	34.4 ± 9.6	
**Gender**		
Male	63	17.5
Female	297	82.5
**Marital status**		
Single	122	33.9
Married	208	57.8
Divorced	9	2.5
Widow	21	5.8
**Level of education**		
Diploma nursing	125	34.7
Associated degree	96	26.7
Bachelor nursing	139	38.6
**Family members numbers**		
< 4	95	26.4
4–6	185	51.4
> 6	80	22.2
Mean ± SD	4.7 ± 2.1	
**Family member with disability**		
Yes	16	4.4
No	344	95.6
**Your husband/ wife**		
Cooperative	156	43.3
Uncooperative	82	22.8
Not applicable (single)	122	33.9
**Your husband/ wife**		
Working	173	48.1
Not working	51	14.2
Irregular work	14	3.9
Not applicable (single)	122	33.9
**Income**		
Enough	214	59.4
Not enough	146	40.6
**Chronic diseases**		
Yes	84	23.3
No	276	76.7
**Workplace is near home**		
Yes	95	26.4
No	265	73.6
**Years of experience in working unit**		
< 5	198	55.0
5–10	45	12.5
10–15	28	7.8
≥ 15	89	24.7
Mean ± SD	9.0 ± 8.2	
**Years of experience in nursing**		
< 5	98	27.2
5–10	88	24.4
10–15	36	10.0
≥ 15	138	38.3
Mean ± SD	12.8 ± 10.7	
**Shifts**		
Morning	125	34.7
Evening	5	1.4
Night	2	0.6
Mixed	83	23.1
8 h shift	4	1.1
10 h shift	12	3.3
12 h shift	112	31.1
> 12 h shift	17	4.7
**Nurse: patient ratio**		
1 - <5 patients	202	56.1
5- <10 patients	136	37.8
> 10 patients	22	6.1
Mean ± SD	15.8 ± 11.7	
**Extra job**		
Yes	97	26.9
No	263	73.1

More than half of nurses (55%) worked for fewer than five years in the working unit, and nearly one fourth (24.7%) had 15 years or more of experience in the working unit, with a mean year of experience of 9 ± 8.2 years. The average years of experience in nursing was 12.8 ± 10.7 years.

representing 38.3% who had 15 years or more of experience in nursing, and slightly more than one-fourth (27.2%) had less than 5 years of experience.

34.7% of the nurses studied worked morning shifts, while only 0.6% worked night shifts. Less than one third (31.1%) reported their work shift was 12 h or more. More than half (55.6%) were assigned to care for 1 to less than 5 patients, and only 6.1% were assigned to 1 to more than 10 patients, with the mean number of beds in a unit of 15.8 ± 11.7 beds. Nearly three-quarters of the nurses studied had no extra jobs (73.1%), whereas 26.9% had extra jobs.

Concerning the number of family members, nearly half (51.4%) of studied nurses had from 4 to 7 Family members, and less than one fourth (22.2%) had more than 6 Family members, with a mean number of family members of 4.7 ± 2.1. Also, 4.4% of nurses’ family members had disabilities. Among married nurses, 21.9% and 20.8% had 2 and 3 children, respectively, while 6.9% didn’t have any children, with a mean of 2.5 ± 0.9 children. More than one-fourth of children (26.4%) are aged 5 to 15 years.

Furthermore, 43.3% of nurses’ partners were cooperative, and 48.1% were workers. Also, it was found that 59.4% of studied nurses had enough income, and nearly one-fourth of studied nurses had chronic disease (23.3%), and 26.4% had a workplace that was near home.

### Correlation between workaholism and work-family conflict among the studied nurses

Table 2 demonstrates that the overall DUWAS and its subdimensions, working excessively and working compulsively, had statistically significant positive and very strong associations (*P* = 0.000, *r* = 0.909; *P* = 0.000, *r* = 0.901), respectively. The total WFC and its subdimensions, work interference with family and family interference with work, both showed a statistically significant positive and strong connection (*P* = 0.000* *r* = 0.911, *P* = 0.000* *r* = 0.886, respectively). Likewise, there was a weak but positive association with the overall WFC (*P* = 0.000*r = 0.41), and a strong and substantial link between the overall OFFER and the overall WFC scale (*P* = 0.000* *r* = 0.53).

**Table 2:** Correlation matrix between workaholism and work-family conflict among the studied nurses (*N* = 360)

Dimensions		Working excessively (WE)	Working compulsively (WC)	Overall DUWAS	Work Interference with Family (WIF)	Family Interference with Work (FIW)
Working excessively (WE)	**r**					
	**p**					
Working compulsively (WC)	**r**	0.634*				
	**p**	< 0.001*				
**Overall DUWAS**	**r**	0.909*	0.901*			
	**p**	< 0.001*	< 0.001*			
Chronic Fatigue	**r**	0.530*	0.424*	0.529*		
	**p**	< 0.001*	< 0.001*	< 0.001*		
Acute Fatigue	**r**	0.383*	0.222*	0.337*		
	**p**	< 0.001*	< 0.001*	< 0.001*		
Inter-shift Recovery	**r**	0.425*	0.317*	0.412*		
	**p**	< 0.001*	< 0.001*	< 0.001*		
**Overall OFER**	**r**	0.566*	0.417*	0.546*		
	**p**	< 0.001*	< 0.001*	< 0.001*		
Work Interference with Family (WIF)	**r**	0.417*	0.333*	0.416*		
	**p**	< 0.001*	< 0.001*	< 0.001*		
Family Interference with Work (FIW)	**r**	0.285*	0.300*	0.324*	0.615*	
	**p**	< 0.001*	< 0.001*	< 0.001*	< 0.001*	
**Overall WFC**	**r**	0.395*	0.353*	0.415*	0.911*	0.886*
	**p**	< 0.001*	< 0.001*	< 0.001*	< 0.001*	< 0.001*

*r* ≥ 0.9 very strong correlation r 0.7-<0.9 strong correlation r 0.5-<0.7 moderate correlation *r* < 0.5 weak correlation * Significant p at ≤ 0.05 high significant *p* ≥ 0.001

### Relation between demographic data and, workaholism, work-family conflict

Table 3 shows that the mean Dutch Work Addiction Scale score for nurses’ professional and sociodemographic attributes, such as age (*p* = 0.440), gender (*p* = 0.273), marital status (*p* = 0.472), years of experience in a working unit (p value = 0.079), and years of nursing experience (p-value = 0.325), does not differ statistically significantly. At the same time, there were notable variations based on shifts (F = 2.8, *P* = 0.007*) and educational attainment (F = 7.3, *P* = 0.001*).

**Table 3:** Workaholism, and work-family conflict according to demographic characteristics of the studied nurses (*N* = 360)

Demographic characteristics	DUWAS	WFC
**Age (years)**		
20-	25.4 ± 5.2	56.3 ± 13.4
30-	25.1 ± 4.8	50.9 ± 14.9
40-	24.4 ± 5.2	46.2 ± 13.3
≥ 50	24.3 ± 4.5	50.2 ± 14.7
**F(p)**	0.903 (0.440)	8.546* (< 0.001*)
**Gender**		
Male	25.7 ± 5.3	52.5 ± 14.2
Female	24.9 ± 4.9	52.1 ± 14.5
**t(p)**	1.099 (0.273)	0.199 (0.842)
**Marital status**		
Single	25.0 ± 4.9	54.7 ± 13.9
Married	24.9 ± 5.2	51.1 ± 14.5
Divorced	26.6 ± 4.9	46.6 ± 17.9
Widow	26.3 ± 3.7	49.7 ± 14.0
**F(p)**	0.841 (0.472)	2.325 (0.075)
**Level of education**		
Diploma nursing	23.7 ± 4.9	47.7 ± 14.1
Associated degree	25.9 ± 5.2	54.9 ± 14.8
Bachelor nursing	25.6 ± 4.8	54.2 ± 13.6
**F(p)**	7.382* (0.001*)	9.541* (0.001*)
**Years of experience in working unit**		
< 5	25.4 ± 5.4	56.6 ± 13.5
5–10	25.6 ± 4.4	53.2 ± 15.1
10–15	23.2 ± 2.8	39.8 ± 10.1
≥ 15	24.4 ± 4.7	45.5 ± 12.5
**F(p)**	2.278 (0.079)	23.203*(< 0.001*)
**Years of experience in nursing**		
< 5	24.5 ± 4.8	54.8 ± 13.8
5–10	25.7 ± 5.0	55.9 ± 13.5
10–15	24.4 ± 6.9	49.5 ± 16.1
≥ 15	25.1 ± 4.5	48.5 ± 14.1
**F(p)**	1.160 (0.325)	6.735* (0.001*)
**Shifts**		
Morning	23.6 ± 4.6	47.3 ± 13.2
Evening	24.6 ± 4.6	54.6 ± 11.3
Night	25.5 ± 3.5	63.5 ± 9.2
Mixed	26.3 ± 5.0	60.8 ± 12.0
8 h shift	23.3 ± 2.1	51.5 ± 9.0
10 h shift	24.9 ± 5.6	59.4 ± 15.4
12 h shift	25.7 ± 5.3	50.0 ± 14.7
> 12 h shift	25.6 ± 4.3	51.9 ± 14.5
**F(p)**	2.840* (0.007*)	8.226* (0.001*)
**Your husband/ wife**		
Cooperative	24.7 ± 4.8	48.5 ± 14.0
Uncooperative	25.7 ± 5.5	55.2 ± 14.7
Not applicable (single)	25.0 ± 4.9	54.7 ± 13.9
**F(p)**	1.122 (0.327)	9.245* (0.001*)
**Your husband/ wife**		
Working	24.4 ± 4.2	49.1 ± 14.0
Not working	26.1 ± 6.7	54.1 ± 15.6
Irregular work	28.9 ± 5.5	59.4 ± 13.9
Not applicable (single)	25.0 ± 4.9	54.7 ± 13.9
**F(p)**	4.494* (0.004*)	5.472* (0.001*)
**Income**		
Enough	22.6 ± 3.6	47.1 ± 12.7
Not enough	28.5 ± 4.7	59.5 ± 13.6
**t(p)**	12.713*(< 0.001*)	8.761*(< 0.001*)
**Chronic diseases**		
Yes	29.0 ± 5.2	59.7 ± 12.9
No	23.8 ± 4.2	49.8 ± 14.1
**t(p)**	8.305*(< 0.001*)	6.019*(< 0.001*)
**Workplace is near home**		
Yes	21.9 ± 4.3	48.7 ± 13.0
No	26.1 ± 4.8	53.4 ± 14.7
**t(p)**	7.917* (< 0.001*)	2.746* (0.006*)

t: Student t-test F: F for ANOVA test, *: Statistically significant at *p* ≤ 0.05 high significant *p* ≥ 0.001

Furthermore, the mean Dutch Work Addiction Scale score for family members with disabilities does not change statistically significantly (F = 0.186, p-value = 0.852). Conversely, the Dutch Work Addiction Scale showed significant differences by nurse: patient ratio (F = 73.6, *P* < 0.001), number of beds in the unit (F = 7.2, *P* < 0.001), mechanical ventilation machines (F = 53, *P* < 0.001), additional job (t = 5.8, *P* < 0.001), number of family members (F = 77, *P* < 0.001), number of children (F = 3.1, *P* = 0.005), and age of children (F = 4, *P* = 0.003).

Additionally, no statistically significant distinction exists between the mean score of the Dutch Work Addiction Scale with husband/ wife cooperation or not (F = 1.122, p value = 0.327). Unlikely there are significant differences in the Dutch Work Addiction Scale according to the husband/ wife occupation (F = 4.4, *P* = 0.004), income (t = 12.7, *P* = 0.004), chronic diseases (t = 8.3, *P* = 0.004*), and how far the workplace is near to home (t = 7.9, *P* < 0.001).

There is an insignificant difference in the overall mean score of the Work-Family Conflict Multidimensional Scale (WFC) among marital status groups (*p* = 0.075). There are unlikely to be significant differences based on age (F = 8.5, *P* < 0.001), sex (F = 0.199, p-value = 0.842), education level (F = 9.5, *P* = 0.001), years of nursing experience (F = 6.7, *P* = 0.001), years of working unit experience (F = 23.2, *P* < 0.001), and shifts (F = 8.2, *P* = 0.001). Furthermore, there is an insignificant difference in the overall mean score of the Work-Family Conflict Multidimensional Scale (WFC) according to family members with a disability (p-value = 0.833). On the other hand, there are significant differences in the overall mean score of the Work-Family Conflict Multidimensional Scale (WFC) regarding nurse: patient ratio (F = 28, *P* < 0.001), number of beds in unit (F = 2, *P* = 0.03), mechanical ventilation machines (F = 99.9, *P* < 0.001), extra job (t = 6.9, *P* < 0.001), family members’ numbers (F = 19.2, *P* < 0.001), number of children (F = 6.2, *P* < 0.001), and age of children(F = 10.9, *P* < 0.001). Additionally, there are significant differences in the overall mean score of the Work-Family Conflict Multidimensional Scale (WFC) regarding the husband/wife corporation (F = 9.2, *P* = 0.001), the husband/ wife occupation (F = 5.4, *P* = 0.001), income (t = 8.7, *P* < 0.001), chronic diseases (t = 6, *P* < 0.001) and how far the workplace is near to home (t = 2.7, *P* = 0.006).

### Linear regression shows the effect of workaholism on Work-Family conflict

Table 4 demonstrates that work-family conflict as a dependent variable and workaholism as an independent variable have a statistically significant regression coefficient value of 17% when employing a linear regression model. This makes the model a significant prediction (F = 74.9; *p* < 0.001). Thus, workaholism has a favorable effect on work-family conflict, where (B = 1.2; *P* < 0.001). This means that for every unit increase in workaholism, there will be a 1.2-point increase in work-family conflict.

**Table 4:** Linear regression shows the effect of workaholism on Work-Family conflict

Variables	B	t	Sig.	95% CI of B	
				LL	UL
Workaholism	1.201	8.658*	< 0.001*	0.928	1.473
R^2^ = 0.173, F = 74.953^*^, *p* < 0.001^*^					

F, p: F and p values for the model

R^2^: Coefficient of determination

B: Unstandardized Coefficients

t: t-test of significance

CI: Confidence interval

LL: Lower limit

UL: Upper Limit

*: Statistically significant at *p* ≤ 0.05

### Multivariate analysis of linear regression between the effect of sociodemographic and professional characteristics on workaholism

Table 5 demonstrates the relationship between personal characteristics and workaholism. Several variables were found to significantly predict workaholism (*p* < 0.05). Nurse-patient ratio (B = 1.613, *p* < 0.001) had the strongest positive effect on workaholism. This indicates that an increase in the number of patients per nurse is strongly associated with higher levels of workaholism. This could be due to the increased workload and stress associated with higher patient loads, leading to compulsive working behavior, number of beds in the unit (B = 0.126, *p* < 0.001), and mechanical ventilation machines (B = 1.503, *p* < 0.001) also showed significant positive associations with workaholism. Larger units with more beds and machines may require more responsibility and attention from nurses, contributing to overwork and workaholic tendencies, and income (B = 2.600, *p* < 0.001) positively predicted workaholism, suggesting that individuals with higher income are more prone to working excessively. This may reflect a drive for financial success or the pressure to maintain a high-income lifestyle.

**Table 5:** Multivariate analysis linear regression between the effect of sociodemographic and professional characteristics on workaholism

Items	B	T	Sig.	95% CI of B	
				LL	UL
Level of education	0.184	0.823	0.411	-0.256	0.625
Shifts	0.083	1.258	0.209	-0.047	0.214
Nurse: patient ratio	1.613	5.157*	< 0.001*	0.998	2.228
Number of beds in unit	0.126	7.722*	< 0.001*	0.094	0.159
Extra job	-0.923	-2.217*	0.027*	-1.742	-0.104
Your husband/ wife / Irregular work	-0.036	-0.194	0.846	-0.406	0.333
Income	2.600	6.558*	< 0.001*	1.821	3.380
Chronic diseases	-1.520	-3.487*	0.001*	-2.377	-0.663
Workplace is near home	1.354	3.345*	0.001*	0.558	2.150
R^2^ = 0.645, F = 52.489^*^, *p* < 0.001^*^					

F, p: f and p values for the model

R^2^: Coefficient of determination

B: Unstandardized Coefficients

t: t-test of significance

CI: Confidence interval

LL: Lower limit

UL: Upper Limit

*: Statistically significant at *p* ≤ 0.05

On the other hand, extra jobs (B = -0.923, *p* = 0.027) had a significant negative relationship with workaholism. Those with additional jobs might distribute their working hours across different positions, reducing the tendency to overwork in any single job, chronic diseases (B = -1.520, *p* = 0.001) were negatively associated with workaholism, which could indicate that individuals suffering from health issues may not be physically or mentally capable of working excessively and, workplace proximity (B = 1.354, *p* = 0.001) positively impacted workaholism, suggesting that those living near their workplace might feel compelled to work longer hours, possibly due to the convenience of access. Interestingly, the level of education, shifts, number of children, age of children, and irregular work schedules of spouses did not significantly predict workaholism, as their p-values were greater than 0.05. The model explains 64.5% of the variance in workaholism (R² = 0.645), and the overall model was statistically significant (F = 52.489, *p* < 0.001). This highlights that the personal characteristics included play an important role in influencing workaholism, although other factors not included in this model may also contribute.

The multivariate linear regression analysis in Table 5 highlights significant relationships between Sociodemographic and professional characteristics and work-family conflict. Several factors emerged as statistically significant predictors, which are worth noting:

Years of experience in the working unit (B = -0.211, *p* = 0.022) had a significant negative effect on work-family conflict. This indicates that individuals with more years of experience in their specific unit report lower levels of work-family conflict. Greater familiarity with the unit’s operations and workflow could enable better time management and reduce the tension between work and family roles. Nurse-patient ratio (B = 2.292, *p* = 0.038) showed a significant positive effect on work-family conflict. An increase in the number of patients per nurse is associated with higher conflict, likely because a higher patient load demands more time and attention, leaving less availability for family responsibilities.

The number of beds in the unit (B = -0.184, *p* = 0.002) had a significant negative relationship with work-family conflict. This counterintuitive result might suggest that in larger units with more beds, there may be better staffing and support, or the increased workload could be mitigated through better processes, reducing the conflict between work and family. Mechanical ventilation machines (B = 5.420, *p* < 0.001) were a significant positive predictor of work-family conflict. Handling mechanical ventilation machines likely adds complexity and stress to the job, leading to an increase in work demands that spill over into family life, thus increasing conflict.

Income (B = 5.972, *p* < 0.001) was another significant positive predictor. Higher-income individuals may face greater job demands, leading to more work-family conflict. This could be due to higher responsibility at work or longer working hours. Chronic diseases (B = -4.339, *p* = 0.007) were negatively associated with work-family conflict. Employees with chronic illnesses may have reduced work expectations or may prioritize their health over excessive work involvement, thereby experiencing less work-family conflict.

Although other factors, such as age, level of education, shifts, and extra jobs, were not statistically significant (*p* > 0.05), some variables approached significance. For instance, having an uncooperative spouse (B = 2.290, *p* = 0.055) showed a near-significant positive relationship with work-family conflict, indicating that spousal support (or lack thereof) might still play a role in conflict levels. The model explains 46.8% of the variance in work-family conflict (R² = 0.468), and the overall regression model is statistically significant (F = 18.838, *p* < 0.001), indicating that demographic variables and personal characteristics are important contributors to work-family conflict. However, it is likely that other unexamined factors also contribute to the overall experience of work-family conflict.

### Linear regression shows the effect of workaholism on work-family conflict

Table 6 shows the connection between workaholism and work-family conflict, showing that both workaholism’s components, working compulsively (WC) and excessively (WE), significantly increase work-family conflict. The effects of working excessively (WE) are very noticeable, with an unstandardized coefficient (B) of 1.434 and a highly significant p-value (< 0.001), suggesting that as individuals engage in excessive work behaviors, the spill over into family life becomes more pronounced. The confidence interval for WE (0.821 to 2.048) further supports the robustness of this finding, as the range does not include zero.

**Table 6:** Linear regression show effect of workaholism on Work-Family conflict

	B	t	Sig.	95% CI of B	
				LL	UL
Working excessively (WE)	1.434	4.596*	< 0.001*	0.821	2.048
Working compulsively (WC)	0.913	2.770*	0.006*	0.265	1.562
R^2^ = 0.174, F = 37.527^*^, *p* < 0.001^*^					

F, p: f and p values for the model

R^2^: Coefficient of determination

B: Unstandardized Coefficients

t: t-test of significance

CI: Confidence interval

LL: Lower limit

UL: Upper Limit

*: Statistically significant at *p* ≤ 0.05

Similarly, working compulsively (WC) is also a significant predictor of work-family conflict, with a coefficient (B) of 0.913 and a p-value of 0.006, indicating that compulsive tendencies, such as feeling psychologically driven to work, exacerbate tensions between professional and personal responsibilities. The confidence interval for WC (0.265 to 1.562) confirms the reliability of this effect. Although the impact of WC is slightly weaker than WE, it remains significant, highlighting that both behavioral and cognitive aspects of workaholism contribute to work-family conflict.

The overall model explains 17.4% of the variance in work-family conflict (R² = 0.174), and the F-statistic (F = 37.527, *p* < 0.001) confirms that the model is statistically significant. While the R² value suggests that other factors also contribute to work-family conflict, this finding emphasizes the critical role of workaholism in influencing this outcome. These results have significant implications for organizations and individuals, suggesting the need for targeted strategies to reduce excessive and compulsive work behaviors, such as promoting healthier work cultures, fostering time management skills, and encouraging employees to prioritize work-life balance. Addressing these behaviors can potentially alleviate work-family conflict, leading to better well-being and productivity.

## Discussion

In the unstable healthcare labor market, employers are increasingly evaluating workers based on their effectiveness and performance. Workers are under more and more pressure to perform well to stand out. Public health should be concerned about workaholism’s possible detrimental effects on health, even though it hasn’t been formally classified as a mental or behavioral condition and is sometimes viewed as a good trait [15–17].

The current study demonstrated that less than one-half of the nurses studied were workaholics and more than one-third were relaxed workers, while a minority of them were compulsive workers. A similar study conducted by Ahmed et al. (2022) focused on a sample population of 300 nurses working in critical care units across major hospitals in Egypt indicated that approximately 70% of the nurses surveyed exhibited workaholic tendencies, and 20% of the sample demonstrated moderate work engagement without excessive or compulsive behaviors [18]. These findings are higher than the current study. On the contrary, the findings of the present study are higher than the results of Borges et al. (2021), which indicated that 27% of workaholic nurses were identified with a higher mean value for excessive work [19].

The findings in the current study could be attributed to workaholism among critical care nurses is a complex issue influenced by both individual and systemic factors. Critical care nursing is inherently demanding, often requiring long shifts, intense patient interaction, and the ability to make rapid decisions under pressure. In Egypt, where the healthcare system faces significant challenges, critical care nurses may feel an overwhelming sense of duty to fill gaps and meet high patient demands, increasing workaholic behaviors. Nurses in this field might struggle to set boundaries between their professional and personal lives, as the nature of their work often blurs these lines. This tendency is compounded by a culture that values resilience and sacrifice in healthcare, making workaholism a common phenomenon that often goes unnoticed or is even tacitly encouraged.

One of the key insights gleaned from the current study findings is the pressures faced by Egyptian nurses, who often work in challenging conditions with limited institutional support. It also underscores the cultural and economic factors contributing to workaholism in Egypt, where high patient loads, a shortage of nursing staff, and societal expectations for healthcare workers to be self-sacrificing are prevalent. The socioeconomic pressures and limited opportunities for career advancement also contribute to the rise of workaholism among critical care nurses. Many nurses work extended hours or multiple jobs to supplement their income, which, in many cases, is insufficient to support their families or meet their financial obligations. The need to secure financial stability can create a cycle where nurses overcommit to work, neglecting their personal lives and well-being.

From another perspective, the present study indicated that there are statistically significant differences between the mean scores of workaholism and nurses’ level of education and/or working shifts. The study found that those who had an associate degree and/or a bachelor’s degree and worked night shifts were more likely to experience higher levels of workaholism than others. Also, the workaholism rate among nurses who were assigned individually to 10 patients each shift, and who had extra jobs. Furthermore, the prevalence of workaholism was reported among nurses who had inadequate income, especially with irregular employment of their husbands, and had chronic diseases.

Similar findings were provided by Shimazu et al. (2021) throughout their study, which was conducted in Japan. They found that a favorable nurse-to-patient ratio, coupled with the availability of well-maintained equipment and facilities, positively influenced workaholism among critical care nurses [20]. In well-resourced environments, nurses reported feeling a heightened sense of responsibility, often leading to extended work hours and an over-commitment to patient care. This study underscores how the quality of healthcare infrastructure can drive workaholic tendencies.

On the contrary, nurses working closer to home and those with a higher income displayed increased workaholic tendencies, as these factors reduced commuting stress and allowed for financial stability, making additional work hours more feasible. However, the same study found that having additional jobs or experiencing chronic health issues negatively impacted workaholism, as time and energy were divided across multiple responsibilities, which limited the ability to overcome a primary job [21].

In the same context, factors like proximity of the workplace to home and adequate income have been shown to have a positive correlation with workaholic tendencies [22]. When nurses work closer to home, the reduced commuting time may facilitate additional shifts, which can lead to an increased workload. Conversely, other studies have shown that holding extra jobs or having chronic health conditions were negatively associated with workaholism. Nurses facing health challenges or working additional jobs might be less inclined to take on extra responsibilities, thereby reducing workaholic behavior [18].

The current study findings suggest that certain workplace conditions and resources can significantly impact nurses’ tendency toward workaholism, while personal and family-related factors appear to have a less consistent influence. A lower nurse-to-patient ratio, a higher number of available beds, and sufficient mechanical ventilation machines likely contribute to a more manageable workload and a sense of job control, which could encourage nurses to take on additional shifts or work extended hours out of a heightened sense of responsibility and commitment.

Additionally, having a shorter commute or a higher income can make it easier for nurses to work longer hours, as reduced travel time and financial stability may enable them to focus more intensively on their jobs without external stressors. Conversely, nurses with extra jobs or chronic health issues may be less inclined to work overtime or take on additional shifts due to physical or time limitations. These results highlight that workaholism among nurses may stem from the structural conditions of their work environment rather than their personal or family circumstances, suggesting that a supportive, well-resourced workplace can encourage work-intensive behaviors.

Recent studies have confirmed that certain personal factors, such as education level, shift types, and family responsibilities (including the number and age of children), have a non-significant effect on workaholism among nurses. For instance, a study by Chen et al. (2022) found no correlation between nurses’ levels of education and their propensity toward workaholic behavior, suggesting that formal qualifications may not directly influence a nurse’s likelihood to overcommit to work [23].

Similarly, research by Johnson and Carter (2021) demonstrated that whether a nurse worked rotating shifts, night shifts, or day shifts had little impact on their tendency to develop workaholic behaviors, challenging the assumption that shift work inherently leads to workaholism. Family responsibilities, such as the number and age of children, were also found to be non-significant, with the same study noting that these factors did not alter the intensity of work involvement for most nurses [24].

From an alternative perspective, the study’s linear regression analysis revealed that the three most important factors that predicted workaholism were gender (specifically women), perception of work as stressful (which contributed 7%), and negative work-family interaction (which contributed 38.3%). Regarding compulsive work, the findings revealed that negative work-family interaction accounted for 22.5%, the perception of work as stressful contributed 3.6% (among those who perceive their work as stressful), and age accounted for 1.7% (notably among younger nurses). Ultimately, the highest predictive values for excessive work were associated with negative work-family interaction, which accounted for 38.3%, the perception of work as stressful, which scored 7.9% (among those who perceive their work as stressful), and gender, which reached 0.7% (specifically women). As a result, the most important predictor of workaholism and its related aspects is a poor work-family connection.

Borges and colleagues (2021) created a stepwise multiple linear regression model to examine the variables that most effectively accounted for workaholism. They considered socio-demographic factors (such as gender, age, marital status, and educational background) and work-related factors (including professional experience duration, geographical location, type of employment contract, work shifts, stress perception, and leisure activities), along with the dimensions of the Work-Home Interaction Scale [19].

The results indicate that workaholism within the nursing field is likely more significantly affected by workplace dynamics and job demands than by personal or familial factors. This suggests that healthcare administrators and policymakers should prioritize interventions that address structural and organizational modifications, such as staffing ratios and resource availability, over those targeting individual traits or scheduling preferences.

Based on the findings of the current study, it is recommended that healthcare administrators focus on maintaining optimal nurse-to-patient ratios and ensuring sufficient medical equipment and facility resources to support nurses effectively. Hospitals should also consider policies that encourage work-life balance, such as flexible scheduling and resources for managing stress, to prevent workaholism from leading to burnout. Furthermore, targeted support for nurses with chronic conditions or multiple job responsibilities may help mitigate the potential for negative health impacts associated with overwork. By prioritizing a balanced and supportive work environment, healthcare institutions can foster sustainable engagement levels, ensuring that nurses remain both committed to their roles and protected from the adverse effects of workaholism.

The current study presented additional findings. It revealed that the incidence of work-family conflict surpassed that of family interference with work (FIW). Furthermore, behavior-based work interference with family (WIF) was found to be more prevalent than both time-based and strain-based interferences. In terms of family interference with work, the time-based, strain-based, and behavior-based subscales exhibited nearly equivalent levels. Overall, strain-based conflicts were more significant than behavior-based conflicts. These results may be linked to the intricate nature of work and the responsibilities faced by nurses in critical care settings. Additionally, time is a crucial element to consider when addressing the needs of patients in these units.

Similar conclusions were drawn in the study conducted by Alazzam et al. (2017). The researchers found that work-family conflicts among nurses in Jordan were more pronounced compared to those faced by nurses in other countries. These results may be linked to several factors, one of which is related to religious beliefs. In Jordan, Islam is the predominant religion, which views the family as a vital and sacred element of society, with each member holding specific rights and responsibilities. The need for attentive care for children and elderly parents is significant. Consequently, nurses, as family members, bear numerous responsibilities towards their children or parents. Furthermore, Islamic and Arabic customs encourage mutual assistance during important occasions such as weddings, mourning, and the preservation of family connections. The nursing profession is inherently demanding and involves a substantial workload, which can hinder individuals’ ability to participate effectively in family and community activities, potentially leading to conflicts between work and family [25].

The results of the present study reveal that younger nurses, especially those holding an associate degree in nursing and possessing limited experience, often encounter conflicts between work and family. This demographic frequently works mixed shifts that exceed 10 h and is tasked with caring for a substantial number of critical patients. Furthermore, nurses who have secondary employment, extensive family responsibilities, and children are likely to struggle with uncooperative partners, particularly if they are non-regular employees with low income and chronic health conditions, in addition to facing long commutes to their places of work, which intensifies their work-family conflicts.

According to the research conducted by Alazzam et al. (2017), nurses face a greater degree of work-to-family conflict compared to family-to-work conflict. This observation may be linked to the economic situation in Jordan, where low family income levels necessitate longer working hours and, in some cases, multiple jobs for parents. These difficult economic conditions require both men and women to participate in the workforce, consequently diminishing the time available for family interactions and adversely affecting family responsibilities, which leads to conflicts between professional and family life. While the authors of the study did not assess the income levels of the nurses, this conclusion is drawn from the researchers’ awareness of the generally low salaries that hospital nurses in Jordan receive, a fact that is widely acknowledged [25]. These conditions parallel the situation in Egypt.

As noted by Alazzam et al. (2017), demographic factors served as independent variables that influenced the variation in work-to-family conflict. There was a significant negative connection between work-to-family conflict and younger nurses, indicating they experienced higher levels of such conflict. Furthermore, female nurses with a greater number of children reported increased levels of work-to-family conflict. The absence of childcare services at the workplace was also positively correlated with work-to-family conflict. Approximately 25.4% of the variance in family-to-work conflict was linked to the collective effect of these independent variables. While the overall model was significant, both age and the youngest child’s age revealed a significant relationship with family-to-work conflict. Within this framework, age exhibited the strongest correlation with family-to-work conflict, suggesting that as nurses grow older, the family-to-work conflict lessens, whereas those with younger children tend to experience higher levels of family-to-work conflict [25].

The results of the current study indicated that the nurse-to-patient ratio and income are significant predictors of overall work-family conflict (WFC). Conversely, factors such as years of experience in the unit, being assigned to a greater number of beds, and the presence of chronic illnesses were found to have a negative correlation with WFC. These results differ somewhat from those reported by Alazzam et al. (2017), who came to the conclusion that work-family conflict was lower among older nurses. Additionally, this study discovered that work-to-family conflict and gender were significantly correlated, with female nurses reporting such problems more frequently than their male counterparts.

The current study’s findings showed that total work-family conflict (WFC) is significantly predicted by both income and the nurse-to-patient ratio. On the other hand, WFC was found to be negatively correlated with factors including years of experience in the present unit, being allocated to a larger number of beds, and having chronic illnesses. These results differ somewhat from those reported by Alazzam et al. (2017), which suggested that older nurses experience lower levels of work-family conflict. Furthermore, this study found a significant association between gender and work-to-family conflict, with female nurses reporting such conflicts more often than their male colleagues [25].

In the authors’ view, one potential explanation for the elevated levels among Egyptian nurses may be the work factors that impede the ability to meet family obligations, including workload, stress from work, scheduling conflicts, shift work, and hours worked. The findings of this study might be attributed to younger nurses who tend to possess less experience, limited decision-making abilities, and a reduced level of autonomy, which can obstruct the resolution of conflicts between work and personal life. Another factor contributing to this outcome could be the nature of the roles of younger nurses, such as those working at the bedside or on mixed shifts, which may provide inadequate and unsuitable time to manage responsibilities in various areas.

Additionally, Alazzam et al. (2017) stated that Jordanian nurses facing family-work conflict often report lower levels of satisfaction when family responsibilities interfere with their job duties due to either work or home circumstances. In fact, both forms of work-family conflict ultimately result in feelings of dissatisfaction, which can adversely impact employee commitment, job performance, quality of care, patient satisfaction, and the desire to leave the job [25].

In contrast to findings from AlAzzam et al. (2017) in Jordan, where female nurses reported considerably higher levels of work-family conflict (WFC), our study did not identify any significant gender differences in WFC [25]. The sociocultural and economic distinctions between Egypt and Jordan could be the cause of this discrepancy. Male and female nurses may feel pressured to prioritize work involvement due to Egypt’s persistent economic hardship, which is characterized by high inflation rates, stagnating pay, and growing living expenses. This could result in higher levels of workaholism for both sexes. Similar WFC experiences could arise from the dilution of conventional gender differences in work and family duties caused by these common economic stressors.

Jordanian society, on the other hand, can still adhere to more traditional gender norms, where women are expected to perform a greater proportion of household chores. This could lead to greater conflict between work and family obligations. These national-level disparities may also be influenced by differences in organizational policies (such as flexible scheduling and childcare help) and larger social support networks. These results underline the need of placing gender dynamics in their economic and cultural contexts and point to the necessity of conducting cross-national research in the Middle East to learn more about how regional circumstances affect work-family balance.

The organization of work varies considerably based on work schedules, which in turn affects work-family conflict (WFC). Nurses on 10- or 12-hour night shifts frequently express dissatisfaction with staff handovers during shift changes. Those on 12-hour day and night shifts, as well as those on alternating shifts that include numerous night shifts, often lack clarity on what information should be communicated to patients or their families. They tend to be more concerned about making errors and report experiencing poor teamwork. Over 30% of day shift nurses exhibit a high level of overcommitment, finding it challenging to disconnect from work at home. Nurses on 12-hour day shifts and those on alternating shifts report facing more interruptions and disturbances at work, along with high demands and significant physical strain. Although nurses on 10- or 12-hour shifts often claim greater satisfaction with their family life, their overall health tends to be poorer compared to those working 8-hour shifts [26].

The primary objective of this research was to evaluate the connection between workaholism and work-family conflict, which was found to be significant. In contrast, the associations between excessive work and compulsive work with family-work conflict were negative, differing from the positive correlation observed between work-family interference and the two dimensions of the Dutch Workaholism Scale (DUWAS). Furthermore, the relationship between work interference with family (WIF) was more pronounced in cases of excessive work compared to compulsive work, which is in opposition to the negative correlation between family interference with work (FIW) and both excessive and compulsive work. The link between overall work-family conflict (WFC) and excessive work is stronger than that with compulsive work, particularly regarding excessive work. The correlations among the studied constructs suggest that workaholism, encompassing both excessive and compulsive work, can effectively predict work-family conflicts among nurses. Additionally, linear regression analysis indicated that the most significant predictors of work-family conflict were workaholism variables, which accounted for 17.3%, followed by occupational fatigue at 13%.

A reasonable interpretation of this relationship forecast is that individuals who are workaholics often display traits associated with perfectionism. Perfectionists typically struggle more with delegating responsibilities, as they believe they are the sole individuals capable of executing specific tasks. Such mindsets can lead to conflicts with colleagues and create strain in their interpersonal relationships. Consequently, these adverse interactions are linked to increased levels of stress [27].

The current research suggests that one potential reason for the identified negative correlation between work-family commitment (WFC) and the incidence of work-family conflict among nurses may be attributed to work-related factors that impede their ability to fulfill family obligations. These factors include workload, stress, scheduling challenges, shift patterns, and working hours. Additionally, nurses who frequently experience family-to-work conflict (FWC) tend to report diminished satisfaction due to family responsibilities interfering with their professional duties, which can stem from either workplace or home conditions. Ultimately, conflicts arising from both work and family spheres can lead to feelings of dissatisfaction, potentially affecting employee commitment, job performance, quality of care, patient satisfaction, and the propensity to leave their positions.

However, the positive correlation between WFC and obsessive work and the negative correlation with family-work conflict can be explained by a depletion of resources because high levels of attachment to work are probably.

to increase nurses’ physical and mental workload, which will limit their ability to contribute to the family domain (e.g., by making them less likely to attend to other family members and take part in family activities).

The research conducted by Zselyke et al. (2024) indicates that both the dimensions of excessive and compulsive work behavior associated with workaholism can serve as predictors for conflicts between work and family, as well as family and work. However, these relationships are characterized by weak correlations, thereby providing a partial answer to the research questions posed. It is suggested that personal and professional demands contribute to an increased perception of workload and fatigue during work hours. Furthermore, the authors contend that a lack of a supportive organizational climate for families, as a resource, can result in collaborative interference between family and work, affecting the dynamics of workaholism, workload, and exhaustion. The findings strongly support the notion that employees exhibiting a heightened workaholic tendency face greater workloads and experience increased work-family conflict [28].

Similar results were also observed by Ruiz-Garcia et al. (2022), who identified a statistical association between workaholism and work-home interaction, noting that workaholics exhibited higher mean scores compared to their non-workaholic counterparts. The study revealed that 28.3% of the participants were classified as workaholics. Furthermore, workaholics consistently demonstrated higher mean scores than non-workaholics, and workaholism was statistically linked to work-home interaction across all four categories. Additionally, workaholism was found to be correlated with perceived work stress [29].

The findings of this study demonstrated how important it is to recognize the resources and demands of both the home and workplace because they have been demonstrated to have an impact on one another. Furthermore, our results imply that occupational health treatments should be used to identify high-risk work profiles, considering the essential role critical care nurses play in the healthcare system.

In the end, there was only a weak statistical association between excessive work and the favorable work-family interaction. In summary, the results showed a comparatively weak correlation between workaholism and both directions of family-work contact. Furthermore, in line with previous studies, a weak and negative connection between workaholism and poor work-family contact was noted [30].

## Conclusion

Workaholism and work-family conflict among critical care nurses were shown to be moderately but statistically significantly correlated in this study. More negative work-to-home and home-to-work interactions were linked to higher levels of workaholism, while more positive interactions in both directions were reported. This suggests a complex dynamic where workaholic behavior may simultaneously structure and strain work-family boundaries. The results validate that workaholism has a significant impact on nurses’ work-family interaction, which answers the main research question. Those with workaholic tendencies reported higher emotional strain, role interference, and a decreased ability to recover outside of work. Critical care nurses frequently face high occupational expectations. These tendencies might raise the risk of burnout, diminish job satisfaction, and increase stress. Future studies should examine these consequences, as the data did not explicitly evaluate outcomes like turnover or the influence on patient care.

In light of these findings, healthcare businesses ought to think about focused tactics, including keeping an eye on excessive work habits, providing organized assistance for integrating work and life, and cultivating organizational cultures that prioritize psychological healing and environmentally friendly work practices. These initiatives could foster healthier, more effective nursing settings and lessen work-family friction.

Moreover, the detrimental effects of workaholism can be lessened by creating an environment at work that values nurses’ mental health and encourages the separation of work and personal life. In addition to improving nurses’ emotional well-being, addressing these problems may also increase staff retention, the standard of patient care, and organizational performance.

### Strengths and limitations

This research focuses on the specific context of nursing in Egypt, providing valuable insights for both regional and local practices. One of its key strengths lies in the robust statistical analysis and large sample sizes, which yield reliable findings regarding the effects of workaholism on nurses’ capacity to maintain equilibrium between their professional obligations and responsibilities to their family. However, the study is not without its limitations. It uses data that is self-reported, which may lead nurses to exaggerate their workaholism-related skills or attitudes, potentially introducing response bias. Furthermore, the dependence on self-reported data raises concerns about biases, including social desirability bias, in which individuals give answers they believe to be positive rather than ones that accurately represent their actual emotions or actions. The conclusions drawn from this study are based on a convenience sampling method rather than a randomized sampling approach, which may restrict the generalizability of the findings due to the non-random selection of the sample, possibly leading to bias. Also, applying the findings to nurses in other hospitals or healthcare settings is difficult because the research’s external validity is limited by its single-hospital setting.

A single-site convenience sample from Alexandria Main University Hospital limited the generalizability of the results, even though this study benefited from a comparatively large sample size (*N* = 360), which was determined by a power analysis using G*Power. Critical care nurses’ experiences in other organizational or cultural contexts, such as those in rural areas, private hospitals, or healthcare systems in other countries like the Gulf or sub-Saharan Africa, may not be adequately represented by the setting’s homogeneity. Both workaholism and the dynamics of work-family conflict may be impacted by these differences. To improve external validity and investigate whether contextual factors result in distinct patterns of association, future research is therefore advised to use multi-site, stratified sampling across various institutional settings.

The study’s only reliance on self-reported data, which is susceptible to common method variance (CMV) and social desirability bias, is another drawback. Participants may have unintentionally inflated or underestimated their behaviors and experiences to conform to socially accepted norms, given the delicate nature of workaholism and work-family conflict. The apparent correlations between the variables might have been overstated as a result. A certain amount of bias is unavoidable in self-report designs, despite efforts to reduce CMV, such as guaranteeing anonymity, using verified tools, and avoiding evaluative language. To validate findings and lessen dependence on subjective views, future study could use multi-source data, such as objective workload indicators or supervisor assessments.

### Implications for nursing practice

Organizations face significant challenges when workaholism becomes normalized within work workplace culture. Although overworking may initially appear to boost productivity, the long-term consequences include reduced efficiency, increased absenteeism, and higher healthcare costs for employees experiencing burnout. Furthermore, a culture that glorifies excessive work can perpetuate unhealthy behaviors, making it harder for staff to maintain boundaries between work and personal life. Organizations must invest in policies and programs to combat these trends, such as flexible working arrangements, wellness initiatives, and leadership practices that prioritize balance and well-being.

At a systemic level, the implications extend to healthcare policy and ethical considerations. Normalizing workaholism risks creating systemic issues that compromise ethical care delivery and staff well-being. Addressing these challenges requires both organizational commitment and broader policy changes, including addressing staffing shortages and ensuring adequate resources for critical care units. By understanding and mitigating the relationship between workaholism and work-family dynamics, healthcare institutions can create healthier work environments, enhance staff satisfaction, and ultimately improve patient care outcomes.

### Future studies

Future research could look at how long-term exposure to workaholism affects clinical reasoning and creative self-efficacy to better understand the long-term effects of workaholism on nursing wellbeing. As nurses have more experience using workaholism and work-family conflict tools, longitudinal research may offer deeper insights into how their attitudes and competencies change over time. Furthermore, studies could investigate how organizational elements like training initiatives, workplace culture, and leadership support influence the response of nurses and the effectiveness of organizational support. Examining the experiences of nurses in various healthcare environments, such as underserved or rural areas, will also assist in identifying potential obstacles to the equitable adoption of AI in various situations. In the end, this would direct the creation of customized plans to optimize AI’s advantages in nursing.

## Data Availability

The corresponding author can provide the datasets created and analyzed for this work upon reasonable request.

## Abbreviations

DUWAS: Dutch Work Addiction Scale
WE: Working excessively
WC: Working compulsively
WFC: Work-Family Conflict Multidimensional Scale
WIF: Work Interference with Family
FIW: Family Interference with Work
